# Sensory-spinal neuron co-culture platform enables analysis of sensory-driven spinal activation

**DOI:** 10.3389/fnins.2025.1619340

**Published:** 2025-07-07

**Authors:** Yuki Miyahara, Kenta Shimba, Kiyoshi Kotani, Yasuhiko Jimbo

**Affiliations:** ^1^Department of Human and Engineered Environmental Studies, Graduate School of Frontier Sciences, The University of Tokyo, Chiba, Japan; ^2^Department of Precision Engineering, School of Engineering, The University of Tokyo, Tokyo, Japan

**Keywords:** co-culture, dorsal root ganglion neuron, spinal neuron, electrical recording, optogenetic stimulation, microfabrication, high-density microelectrode array

## Abstract

**Background:**

Pain plays a crucial role in selecting behaviors essential for survival. Nociceptive stimuli are converted into neuronal signals by dorsal root ganglion (DRG) neurons and transmitted via the spinal cord to the brain, where pain is perceived. Chronic pain, characterized by prolonged nociceptive signaling, significantly reduces the quality of life. Specifically, nociplastic pain arises due to heightened spinal neuronal activity. However, the mechanisms underlying this persistent increase remain unclear, impeding the development of effective treatments. Therefore, the present study aimed to develop an experimental platform to investigate how sensory neuron signals increase spinal neuronal activity.

**Methods:**

We developed a specialized microstructure enabling a separate culture of DRG and spinal neurons connected functionally by axons extending through microtunnels.

**Results:**

Immunofluorescence staining confirmed precise spatial separation and robust neuronal network formation. Microstructures were integrated with high-density microelectrode arrays to facilitate electrophysiological recordings during co-culture. Optogenetic stimulation of DRG neurons significantly activate the spinal neurons, which are not active spontaneously, and increase synchronous activity by 11.8-fold in the spinal neuronal network. Notably, elevated spinal neuron activity persisted for at least 20 min after stimulation ceased, indicating a prolonged neuronal response.

**Conclusion:**

This novel co-culture system provides a powerful tool for elucidating the pathogenic mechanisms underlying chronic pain, potentially guiding future therapeutic strategies.

## 1 Introduction

Pain serves an important biological function by guiding behaviors that enhance survival likelihood (D’Mello and Dickenson, 2008). Organisms perceive nociceptive stimuli and tissue damage as pain, which prompts behaviors such as avoidance of harmful stimuli or resting for recovery. Nociceptive stimuli are converted into neuronal signals by dorsal root ganglion (DRG) neurons. These signals are subsequently transmitted via the spinal cord to the brain, where they are consciously perceived as pain.

Chronic pain is characterized by persistent pain perception even in the absence of ongoing stimuli, significantly reducing the quality of life of patients. DRG and spinal neurons comprise various cell types that form specific synaptic connections (Sullivan and Sdrulla, 2022). Under normal conditions, neuronal activity originating in DRG neurons is modulated by inhibitory spinal neurons, ensuring appropriate intensity of pain signals reaching the brain (Braz et al., 2014). However, excessive neuronal activity transmitted from DRG neurons to spinal neurons via glutamate and peptides, such as calcitonin gene-related peptide (CGRP), increases excitatory neuronal activity and synchronizes network activity within the spinal cord, causing heightened transmission to the brain (Kuner and Flor, 2017; Jones et al., 2025). Increased frequency of neuronal activity transmitted from the spinal cord to the brain alters neuronal processing in the thalamus and cortex. Pain signals generated in these brain regions result in the chronic pain condition known as nociplastic pain (Haroutounian et al., 2014; Cohen et al., 2021).

Pharmacological therapies commonly used for chronic pain primarily aim to facilitate tissue healing and reduce pain severity (Wylde et al., 2011; Finnerup et al., 2015). However, these treatments have limited efficacy for nociplastic pain. Patients with nociplastic pain lack clear tissue-based sources of pain. In addition, pharmacologically selective treatment of pain-producing thalamic and cortical regions is extremely challenging. Inappropriate pharmacological interventions can potentially lead to drug abuse and dependence (Chang et al., 2015). Notably, compared to the thalamic and cortical regions, the spinal cord is more accessible for intervention. Therefore, it is crucial to develop treatments that can prevent increased spinal neuronal activity before long-term functional changes occur in the thalamus and cortex, potentially halting the progression to nociplastic pain. Such treatments require understanding the mechanisms underlying the sustained increase in spinal neuronal signaling and network activity induced by signal transmission from DRG to spinal neurons.

Both *in vivo* and *in vitro* studies have been conducted to elucidate the mechanisms underlying sustained increases in spinal neuronal activity. Animal model studies (Detloff et al., 2014) and experiments using spinal cord slices (Alles et al., 2021) have indicated that elevated spinal neuronal activity can influence neuronal network function and animal behavior. However, the precise mechanisms by which sensory input from DRG neurons triggers increased spinal neuronal activity remain unclear. To investigate these mechanisms, previous studies have utilized mixed neuronal cultures (Ohshiro et al., 2007) and polydimethylsiloxane (PDMS) microfluidic co-culture systems (Wijdeven et al., 2018; Vysokov et al., 2019). Mix-culture approach successfully reproduced activity transmission from capsaicin-responsive DRG neurons to spinal neurons. However, the specific contribution of DRG neuronal activity transmission could not be evaluated, as some spinal neurons respond directly to capsaicin via its receptor. Notably, the microfluidic co-culture approach allows selective pharmacological stimulation of DRG neurons to induce neuronal activity and evaluate its transmission to spinal neurons. Despite its efficacy, previous co-culture studies have not assessed the relationship between activity transmission from individual subtypes of DRG neurons and the resultant changes in spinal neuronal firing rate. Furthermore, previous recording methods are limited by insufficient temporal or spatial resolution, precluding simultaneous analysis of neuronal activity at single-cell and network levels. Therefore, to elucidate the mechanisms underlying sustained spinal neuronal hyperactivity, an advanced co-culture system enabling single-cell stimulation and simultaneous network-wide recording of individual neuronal activity is required.

Microelectrode arrays (MEAs) have recently been employed to simultaneously record neuronal activity across numerous neurons. Different neuronal types on MEAs have successfully been co-cultured using PDMS microstructures (Shimba et al., 2014). High-density microelectrode arrays (HD-MEAs) provide detailed recordings of individual neurons and neuronal networks (Bakkum et al., 2013; Sharf et al., 2022; Kobayashi et al., 2024). Their high electrode density enables the simultaneous network-wide recording of individual neuronal activity with high spatial resolution (Müller et al., 2015). Additionally, HD-MEAs can be integrated with PDMS microstructures to facilitate neuronal co-culture (Duru et al., 2023). The combination of HD-MEAs and optogenetics (Shimba et al., 2022) enables precise evaluation of neuronal activity induced by the optical stimulation of individual neurons within a network (Kobayashi et al., 2024). Therefore, it is important to establish a method for co-culturing DRG and spinal neurons on HD-MEAs and recording the activity transmitted from DRG neurons to spinal neurons through optical stimulation at both cellular and network levels. Such an approach could provide crucial insights into the mechanisms underlying sustained increases in spinal neuronal activity.

**Figure 1:**
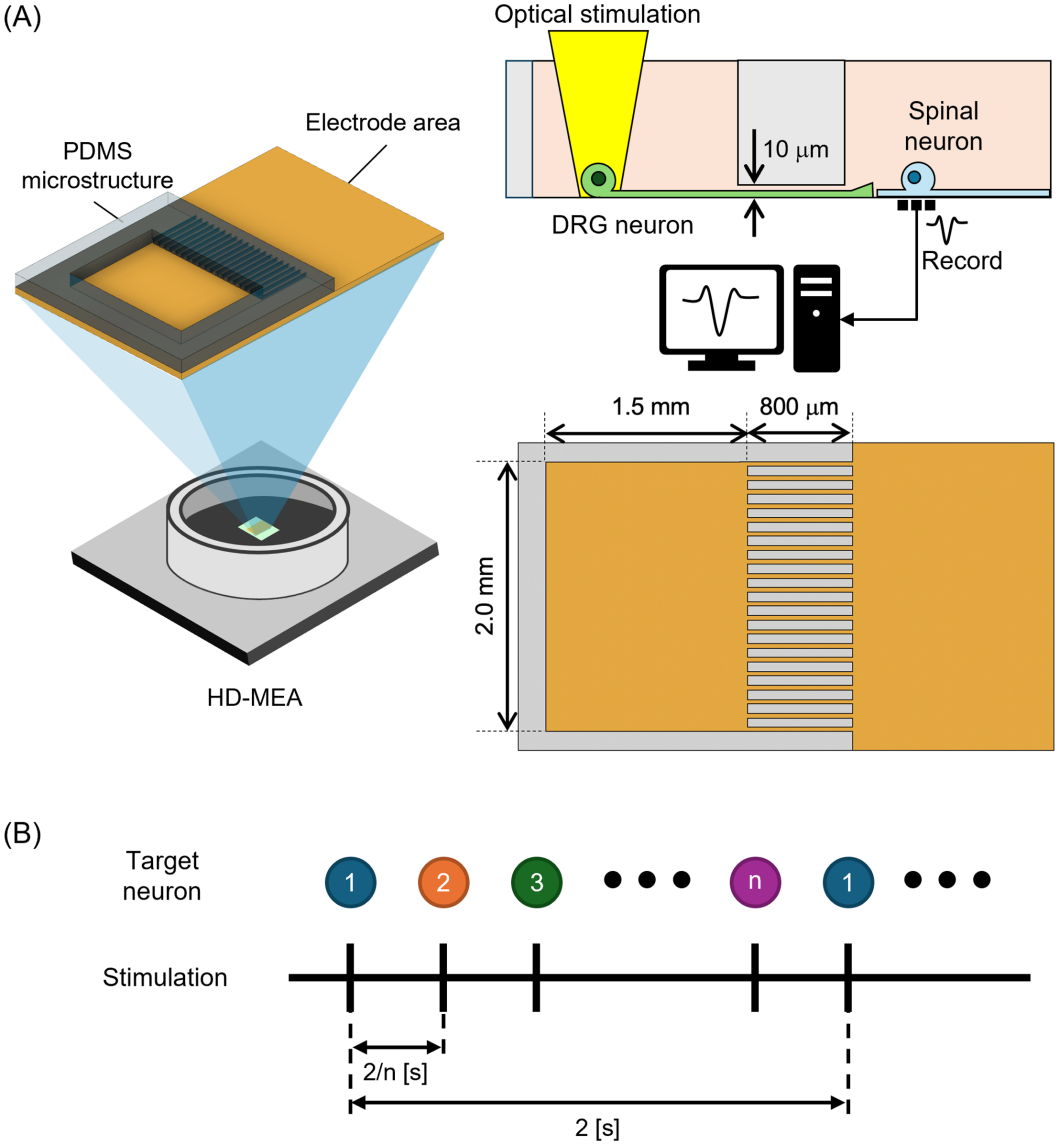
Overview of the *in vitro* platform for co-culturing dorsal root ganglion (DRG) neurons and spinal neurons on high-density microelectrode arrays (HD-MEAs). **(A)** PDMS microstructures, consisting of a culture chamber and microtunnels, were placed onto the HD-MEAs. DRG neurons were cultured inside the chamber, whereas spinal neurons were cultured outside the microstructure. Neuronal activity was induced by individually stimulating neurons expressing Channelrhodopsin-2 (ChR2). Spinal neuronal activity was recorded before, during, and after stimulation. **(B)** Optical stimulation procedure. Optical stimulation pulse was delivered to individual neurons sequentially.

The present study aimed to develop an experimental system to investigate spinal neuronal activity modulation by co-culturing DRG and spinal neurons. Figure 1 presents the overview of the *in vitro* platform for co-culturing neurons on HD-MEAs. DRG and spinal neurons were spatially separated using PDMS microstructures placed on HD-MEAs, and optical stimulation was employed to control activity transmission and recorded the activity of spinal neurons (Figure 1A). Individual neurons were stimulated optically (Figure 1B). Initially, DRG and spinal neurons were spatially separated using PDMS microstructures to establish a co-culture system. Immunofluorescence staining confirmed that DRG and spinal neurons could be cultured separately and that axonal extensions formed a sensory network on the culture dish. Subsequently, DRG and spinal neurons were co-cultured on HD-MEAs, and optical stimulation of DRG neurons resulted in a measurable increase in spinal neuronal firing rate. The proposed co-culture system provides a valuable platform for studying sensory neuron-driven modulation of spinal networks, with implications for the mechanism of chronic pain.

## 2 Materials and methods

### 2.1 Microfabrication

Molds were fabricated using photolithography to process SU-8 photoresist on silicon wafers. The parameters for spin-coating are described in Supplementary Figure 1. Organic contaminants on silicon wafers were removed using acetone (FUJIFILM Wako Pure Chemical Corp., Osaka, Japan), followed by rinsing with isopropanol (FUJIFILM Wako Pure Chemical Corp.) and ultrapure water. After water removal, wafers were completely dried by heating at 100°C for 15 min, followed by plasma treatment to render the surfaces hydrophilic. Hexamethyldisilazane (FUJIFILM Wako Pure Chemical Corp.) was spin-coated onto wafers. Wafers were heated at 95°C for 2 min. Subsequently, SU-8 3005 (Nippon Kayaku Co. Ltd., Tokyo, Japan) was spin-coated onto wafers at a thickness of 10 μm, which were heated again at 95°C for 3 min. A microtunnel template pattern (Supplementary Figure 2A) comprising 20 rectangular lines (each 20 μm wide and 900 μm long, spaced with an 80 μm gap) was created using a maskless exposure system (PALET; NEOARK Corp., Tokyo, Japan). Following exposure, wafers were heated again at 65°C for 1 min and 95°C for 2 min and cooled at room temperature (RT, 21°C - 24°C) for 5 min. A second layer of SU-8 3050 (Nippon Kayaku Co. Ltd.) was spin-coated at a thickness of 50 μm. Wafers were then heated at 95°C for 15 min, cooled at RT for 5 min, and exposed again using PALET to form the culture chamber template pattern (Supplementary Figure 2B). This pattern comprised two rectangular chambers measuring 2 mm wide and 1.5 mm long, spaced 2.3 mm apart. After the second exposure, wafers were heated at 95°C for another 15 min and cooled at RT for 5 min. The exposed SU-8 patterns were then developed for 9 min using an SU-8 developer solution (Kayaku Advanced Materials Inc., Westborough, MA, USA). Following development, wafers were briefly rinsed for 10 s with fresh SU-8 developer and then immersed in isopropanol to confirm the removal of unexposed SU-8. After a final rinse with ultrapure water and removal of water droplets, wafers were heated at 150°C for 15 min.

To fabricate the PDMS microstructures, PDMS was cast onto the prepared SU-8 molds. To prevent the detachment of the SU-8 patterns from wafers during peeling, a Sigma coat (Sigma-Aldrich, St. Louis, MO, USA) was first applied to the molds and allowed to evaporate at RT for 5 min. PDMS base material (SYLGARD 184 Silicone Elastomer Base; Dow Inc., Midland, MI, USA) was mixed thoroughly with the curing agent (SYLGARD 184 Silicone Elastomer Curing Agent; Dow) at a 10:1 weight ratio and poured into molds. After the air bubbles were removed under vacuum, PDMS was cured at 80°C for 1 h. The cured PDMS was carefully peeled from the molds, and the DRG neuron culture chamber was precisely cut out with a scalpel. PDMS microstructures were washed three times with 70% ethanol (FUJIFILM Wako Pure Chemical Corp.) to remove dust, followed by three rinses with ultrapure water and sterilization. To prevent unwanted cell adhesion within the microtunnel walls, PDMS surfaces were coated with bovine serum albumin (BSA; Sigma-Aldrich). Following the protocol described by Tanaka et al. (2016), microtunnels were filled with 3% (w/v) BSA diluted in phosphate-buffered saline (PBS) and incubated overnight at 4°C. Finally, the PDMS microstructures were rinsed once with sterile water, and residual surface water was carefully removed.

### 2.2 Cell culture

DRG and spinal neurons were co-cultured using PDMS microstructures placed on HD-MEA (Maxwell Biosystems AG, Zurich, Switzerland). DRG and spinal neurons were cultured in separate compartments of the PDMS structures.

The HD-MEA MaxOne+ chips were pretreated and coated by following the Maxwell Biosystems protocol (MaxWell Biosystems, 2023). Chips underwent hydrophilic treatment, sterilization by soaking in 70% ethanol for 30 min, washing with ultrapure water, and drying. A 0.1% polyethyleneimine (PEI; Sigma-Aldrich) solution was applied to the electrode surface, and the chips were incubated overnight at 4°C. The electrodes were then washed thrice with sterile water, dried, and subsequently fitted with PDMS microstructures. Laminin (20 μg/ml; Thermo Fisher Scientific, Waltham, MA, USA) was added, with 5 μl applied to the DRG neuron culture chamber and 30 μl to the culture area outside the microstructure. Microtunnels were filled with the laminin solution, and the chips were incubated at 37°C for at least 1 h.

To evaluate whether DRG and spinal neurons could be cultured separately, both neuron types were co-cultured on 35 mm culture dishes (Techno Plastic Products AG, Trasadingen, Switzerland) and cultured individually. For the co-culture condition, culture dishes were prepared in the same manner as in the MaxOne+ chips, and the culture surface was coated with laminin. For the monoculture condition, the PEI-coated surface was washed, dried, and coated with laminin.

DRG and spinal neurons were obtained from 15-day-old Wistar rat embryos (Charles River Laboratory Japan, Inc., Kanagawa, Japan). To isolate DRG neurons, dorsal root ganglia were incubated in 0.25% trypsin solution diluted in Hanks’ balanced salt solution (FUJIFILM Wako Pure Chemical Corp.) and enzymatically dissociated by shaking at 37°C for 14 min. Trypsin treatment was stopped, and DRG neurons were manually isolated. The DRG neurons were seeded at a density of 500 cells/mm^2^ within their designated chambers. Spinal neurons were isolated from embryonic spinal cords and seeded at a density of 3,000 cells/mm^2^ in the culture area outside the PDMS microstructure. After seeding, the cells were incubated for at least 60 min before adding 800 μl of preheated (37°C) medium to the MaxOne+ chip and 2 ml to the 35 mm culture dish.

Animal experiments were conducted following the University of Tokyo Animal Experiment Manual and were approved by the University of Tokyo Animal Experiment Committee (approval number: A2023FS006). The culture medium consisted of Neurobasal Plus medium (Thermo Fisher Scientific) supplemented with 2% B-27 Plus supplement (Thermo Fisher Scientific), 0.5 mM GlutaMAX (Thermo Fisher Scientific), 100 units/ml penicillin, 100 μg/ml streptomycin (Thermo Fisher Scientific), and 50 ng/ml nerve growth factor (FUJIFILM Wako Pure Chemical Corp.). On the fourth day of culture, cells were transfected with an adeno-associated virus (AAV) [pAAV-Syn-ChR2 (H134R)-GFP gifted by Edward Boyden, Massachusetts Institute of Technology, MA, USA; Addgene #58880-AAV8] (Boyden et al., 2005) encoding channelrhodopsin-2 (ChR2), a light-sensitive protein, and green fluorescent protein (GFP). AAV particles were added to the cells at 1.0 × 10^5^ multiplicity of infection. The medium was partially replaced every 3 days, beginning on day 7 of incubation.

### 2.3 Activity recording and optical stimulation experiments

The MaxOne recording system (Maxwell Biosystems) was used to record neuronal activity. The HD-MEAs in this system simultaneously recorded extracellular potentials from up to 1,020 of the 26,400 available electrodes.

Spinal neuronal activity was recorded in response to optical stimulation of neurons under three experimental conditions: “control,” in which no optical stimulation was applied; “DRG stim,” in which DRG neurons were stimulated; and “SC stim,” in which spinal neurons were stimulated. The locations of GFP-expressing DRG and spinal neurons were identified through fluorescence imaging. For accurate spike sorting with HD-MEAs, we selected recording electrodes in units to record the activity of individual neurons while monitoring the overall neural network activity on the HD-MEA. Nine electrodes were grouped into one recording unit, and 110–113 units were selected beneath spinal neurons. Because spinal neurons have low firing rates, it was difficult to use the conventional activity scan method, which relies on an initial screening process that assesses the activity of each electrode to identify those capable of detecting neuronal signals. To select recording electrodes, we employed a fluorescence imaging–based method (Miyahara et al., 2023). Stimulating neurons were chosen based on fluorescence images, and optical stimulation (470 nm wavelength, 50 ×  50 μm square pattern) was applied to individual neurons for 5 ms at a frequency of 0.2 Hz. Spinal neuron activity was recorded under the following sequence: 20 min of spontaneous activity recording, 30 min of activity recording during optical stimulation, and another 20 min of spontaneous activity recording. A total of eight samples were used for the “DRG stim”, whereas two samples were used for the “control” and “SC stim” each. Eight samples cultured for more than 28 days were used in the experiment. When the same samples were used across multiple conditions, experiments were spaced at least 1 week apart to minimize carryover effects.

### 2.4 Data analysis

**Figure 2:**
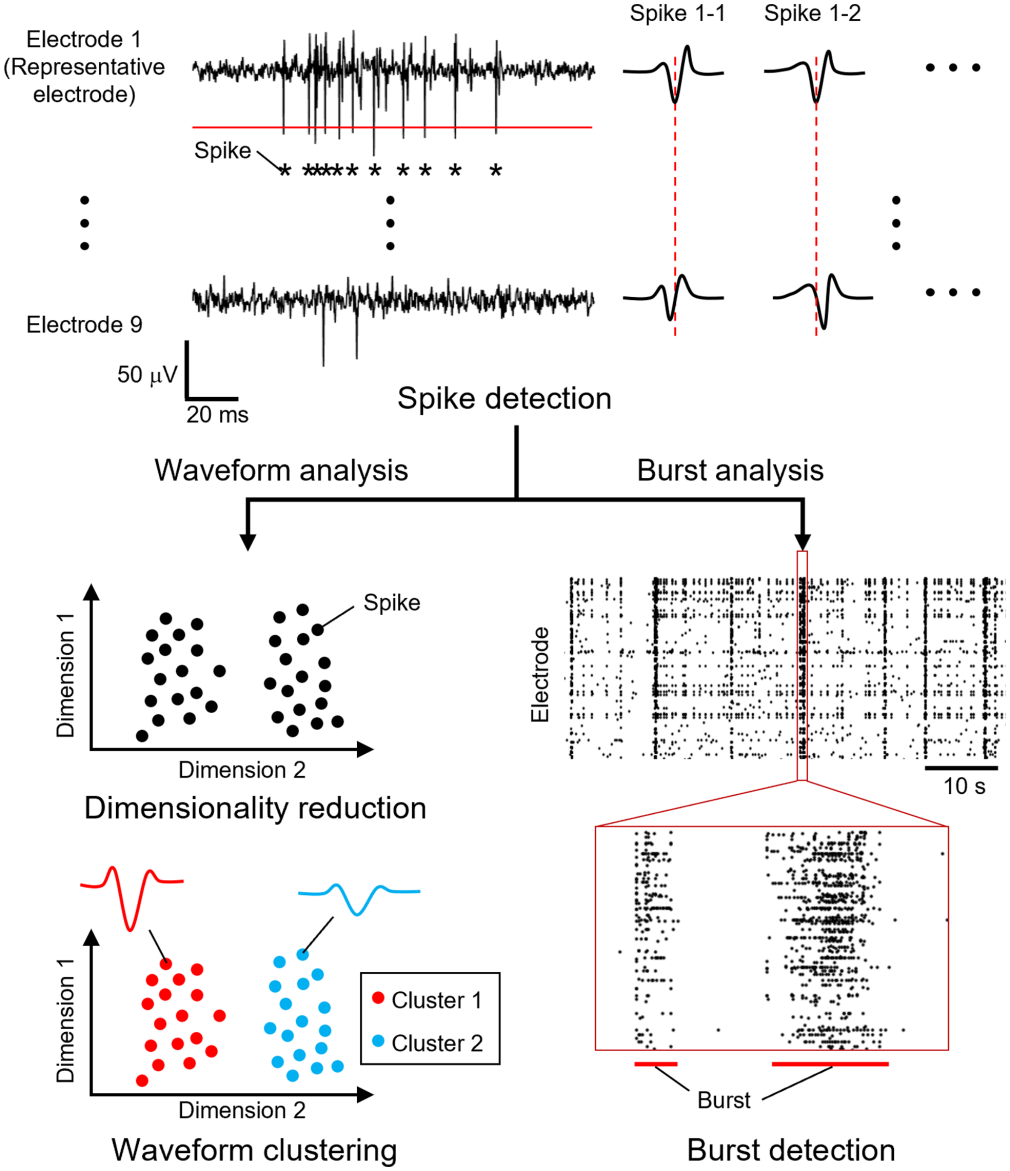
Overview of the data analysis. From a recorded signal, spike times were determined by identifying when the negative peak exceeded a threshold from a selected electrode (red solid line). Within each recording electrode unit, the electrode that recorded the greatest number of spikes was selected as the representative electrode. A total of 41 data points (2 ms) were extracted from each electrode within a recording unit, centered on the detected spike time of the representative electrode (red dotted line). The spike waveform data were reduced to two dimensions, and the projected data were clustered. Bursts were detected as synchronous neuronal activity in multiple neurons.

Spikes and synchronized bursts were detected in the recorded signals (Figure 2). Extracellular recordings from all electrodes were sampled at 20 kHz and processed using a bandpass filter (100–3,000 Hz) to remove low- and high-frequency noise, including power supply interference, while preserving waveforms of neuronal signals. The mean and median absolute deviation of the filtered signal were calculated, and spike times were determined by identifying when the negative peak exceeded a threshold. To determine the spike detection threshold, the standard deviation was estimated as the median of the absolute deviation divided by 0.6745 as previously described (Takekawa et al., 2010), and a threshold was set at five times the estimated standard deviation plus the mean for each electrode. If multiple spikes were detected within 2 ms, only the first spike was retained to account for the neuronal refractory period.

Optically stimulated neurons on the MaxOne+ chip exhibited stimulation artifacts in the recorded data. These artifacts resulted in a monotonically increasing or decreasing reference potential during stimulation application, causing abrupt changes when the stimulation was switched. These changes led to peaks that could be erroneously detected as spikes in the filtered data. To eliminate stimulation-induced artifacts, spikes detected within the time window from 1 ms before the stimulation onset to 1 ms after the stimulation end were excluded from the analysis.

Spikes were clustered into units based on their waveforms. Within each recording electrode unit, the electrode that recorded the greatest number of spikes was selected as the representative electrode. A total of 41 data points (2 ms) were extracted from each electrode within a recording unit, centered on the detected spike time of the representative electrode. The extracted waveforms were sorted into single-waveform clusters according to a previously described method (Lee et al., 2021). To reduce dimensionality, the waveform data were mapped to two dimensions using uniform manifold approximation and projection (UMAP). The projected data were clustered using the density-based spatial clustering of applications with noise (DBSCAN) method. For UMAP dimensionality reduction, the number of nearest neighbors was set to 20, and the minimum distance between data points was set to 0.2. For DBSCAN clustering, the neighborhood radius was set to the value at which the nearest-neighbor distance of each data point increased rapidly, and the minimum number of samples was set to 5. Clusters containing 50 or fewer spikes were excluded from the following analyses. The frequency of neuronal activity was calculated before, during, and after stimulation.

Bursts, defined as the simultaneous activation of multiple neurons, were analyzed as indicators of neuronal network activity levels. Burst detection was performed following the method described by Bakkum et al. (2014). A waveform cluster was classified as a burst if more than 50 spikes occurred within a 100 ms window. The initiation point of each burst was identified using the burst onset time and the spike times of individual waveform clusters. The first spike occurring after the burst onset was considered the activity that triggered the burst, and this burst-triggering spike was determined for each burst. By mapping the corresponding electrode unit location, the burst initiation points were determined. For further analysis, only waveform clusters that served as initiation points in three or more bursts were included.

To quantify the change in neuronal firing rate across experimental conditions, the number of spikes per waveform cluster was calculated for pre-stimulation, stimulation, and post-stimulation periods. Firing rate was estimated by dividing the number of spikes by the recording duration. The relative changes in firing rate during and after stimulation were then compared to baseline (activity levels of before stimulation).

The standard deviation is calculated using the following formula:


$$\mathrm{SD}=\sqrt{\frac{1}{N-1}\sum_{i=1}^{N}{\left(x_{i}-\bar{x}\right)}^{2}}$$

Where $x_{i}$ represents the i -th individual data point, $\bar{x}$ is the mean of the data, N is the total number of data points, and SD is the standard deviation. In the following text, data are presented as mean ± standard deviation.

### 2.5 Immunofluorescence staining

Immunofluorescence staining was performed on three samples of neurons that were either monocultured or co-cultured in 35 mm culture dishes and on five samples used exclusively for the “DRG stim” in the optical stimulation experiment. Cells were fixed with 4% paraformaldehyde (FUJIFILM Wako Pure Chemical Corp.) for 30 min at RT. After washing the samples with PBS (Thermo Fisher Scientific), a blocking solution containing 4% Block Ace (Sumitomo Dainippon Pharma, Osaka, Japan) and 0.25% Triton X-100 (Merck, Darmstadt, Germany) diluted in PBS was applied, and the samples were incubated for 2 h at RT. Primary antibodies were diluted in 0.4% Block Ace and 0.25% Triton X-100 in PBS, added to the samples, and incubated overnight at 4°C. The samples were then washed thrice with PBS for 5 min, followed by the application of secondary antibodies and incubation at RT for 4 h. After a final wash with PBS (three times, 5 min each), fluorescence imaging was performed using a fluorescence microscope. Anti-Peripherin antibody (rabbit, 1:250; Abcam, Cambridge, UK), anti-NeuN antibody (chicken, 1:2,000; Merck), anti-CGRP antibody (mouse, 1:200; Abcam) and anti-Beta 3 tubulin (B3T) antibody (rabbit, 1:500; Abcam) were used as primary antibodies, while Alexa Fluor 488 anti-mouse IgG (goat, 1:500; Thermo Fisher Scientific), Alexa Fluor 546 anti-rabbit IgG (goat, 1:500; Thermo Fisher Scientific) and Alexa Fluor 647 anti-chicken IgY (goat, 1:500; Abcam) antibodies were used as secondary antibodies.

The presence of DRG neurons was determined based on fluorescence imaging, and the percentage of DRG neurons inside and outside the culture chamber of the PDMS microstructure was calculated. Following a previous study (Bulusu et al., 2017), FastPeakFind (version 1.7) was applied to NeuN+ areas to identify peaks in fluorescence intensity, which were used to estimate the number of cultured neurons. Similarly, the number of DRG neurons was determined from Peripherin+ areas. The spatial distribution of DRG neurons was analyzed by superimposing the detection points and calculating the proportion of neuron nuclei that were within 10 μm of a DRG neuron relative to the total number of neuron nuclei. If multiple neuron nuclei were within 10 μm of a DRG neuron, they were counted as a single overlapping point. In the monoculture samples, more than 1,000 neuronal nuclei were identified in each of the three samples. In the co-culture samples, over 100 neuronal nuclei were identified within the culture chamber, whereas more than 1,000 neuronal nuclei were detected outside the PDMS microstructure.

The number of spinal neuron nuclei on the recording electrode units was quantified using immunofluorescence staining in co-cultured DRG and spinal neuron samples grown on HD-MEAs. NeuN+ areas were overlaid with the positions of the recording electrode units to perform the counting. Five samples were used for analysis.

## 3 Results

### 3.1 Separating neurons in the co-culture system

Protein expression analysis through immunofluorescence staining confirmed that DRG and spinal neurons could be successfully separated using the proposed co-culture method. To assess neuron separation, we compared the ratio of DRG neurons to the total number of neuronal nuclei in both monoculture and co-culture samples of DRG and spinal neurons. First, we determined the percentages of DRG and spinal neurons under monoculture conditions. We then evaluated the proportion of DRG neurons under the co-culture condition.

**Figure 3:**
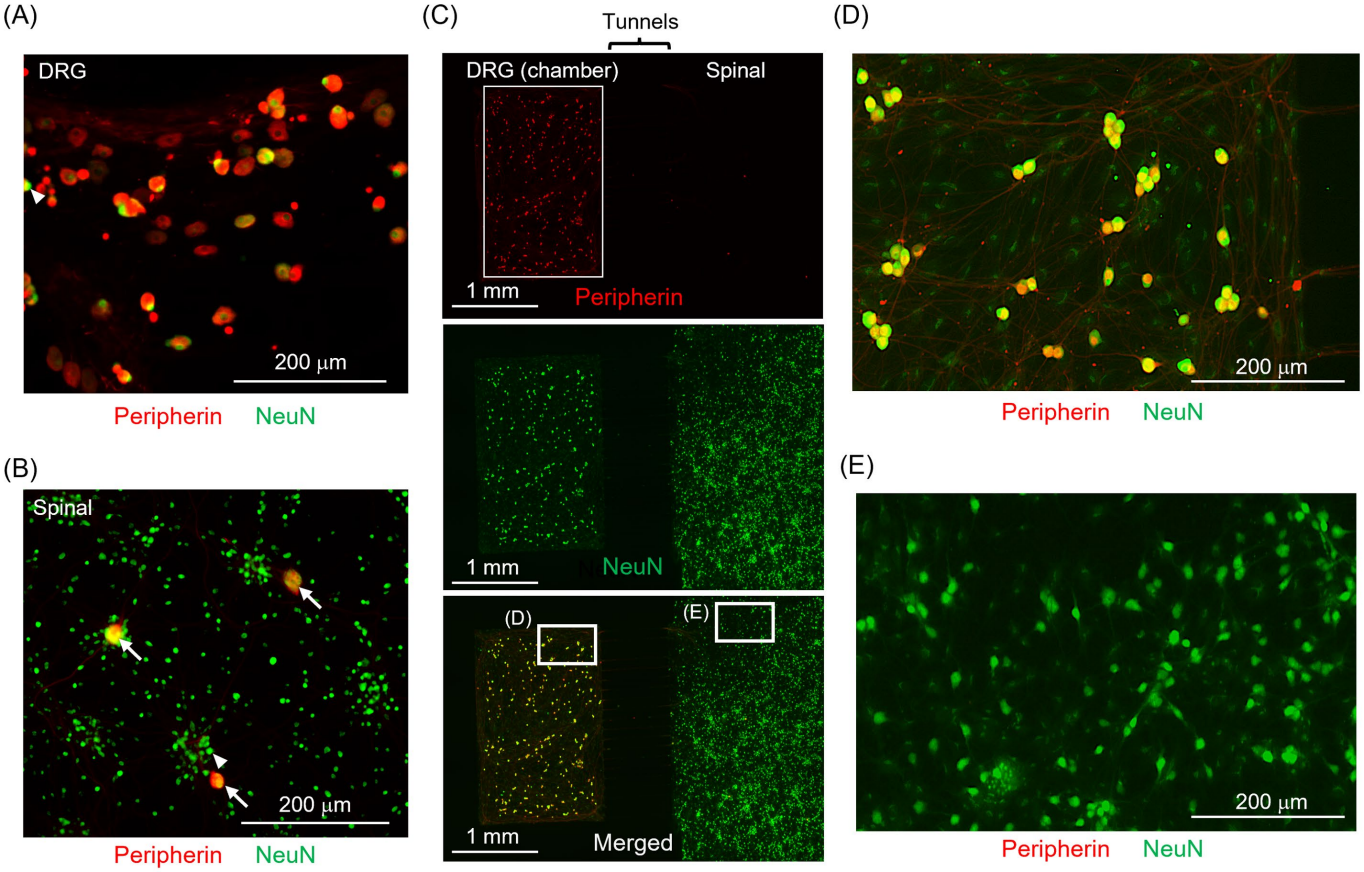
Immunofluorescence staining of DRG neurons and spinal neurons. **(A,B)** DRG and spinal neurons immunofluorescent stained in the monoculture. **(C)** Immunofluorescence staining of co-cultured DRG (left side) and spinal neurons (right side). **(D,E)** Enlarged views of the white-framed areas in **(C)**. Peripherin+ neurons are indicated in red, and NeuN+ neurons in green. White arrows indicate Peripherin+/NeuN+ neurons, whereas white triangles indicate Peripherin−/NeuN+ neurons. In the spinal neuron sample, most neurons are NeuN+ only, whereas in the DRG neuron sample, many neurons are both Peripherin+ and NeuN+. In the co-cultured sample, most neurons within the culture chamber are Peripherin+/NeuN+, whereas most neurons outside the microstructure are Peripherin−/NeuN+. The proposed co-culture method effectively separated DRG neurons from spinal neurons.

Figure 3 depicts the immunofluorescence staining results for DRG neuron and spinal neurons. Peripherin+ areas are indicated in red, and NeuN+ areas are presented in green. White arrows indicate Peripherin+/NeuN+ neurons, whereas white triangles indicate Peripherin−/NeuN+ neurons. In monoculture samples, most DRG neurons were Peripherin+/NeuN+ (Figure 3A), whereas most spinal neurons were Peripherin−/NeuN+ (Figure 3B). Figure 3C presents the results for a co-culture sample. Figures 3D,E each present an enlarged view of the white-framed area in Figure 3C. Most neurons within the culture chamber are Peripherin+/NeuN+ (Figure 3D), whereas most neurons outside the microstructure are Peripherin−/NeuN+ (Figure 3E). Supplementary Figure 3 displays the method to identify Peripherin+/NeuN+ neurons. The percentage of Peripherin+ neurons in the spinal neuron monoculture sample was 0.1 ± 0.1%, whereas that in the DRG neuron monoculture sample was 95.5 ± 1.8% (n > 1,000 neurons for both groups). This result confirms minimal DRG neuron contamination during tissue collection. In the co-culture samples, the percentage of Peripherin+ neurons were 0.3 ± 0.2% outside the microstructures (n > 1,000 neurons) and 94 ± 3% within the culture chambers (n > 100 neurons). There was no significant difference in the percentage of DRG neurons outside the microstructures compared to the monoculture condition. This finding confirms that the proposed co-culture method effectively separated DRG neurons from spinal neurons. In addition, 10.8% of the cultured DRG neurons expressed CGRP (Supplementary Figure 4), which is a pain-related neurotransmitter that can depolarize the membrane potential of spinal neurons. This suggests that pain-related DRG neurons maintained the capability to enhance spinal neuron activity.

**Figure 4:**
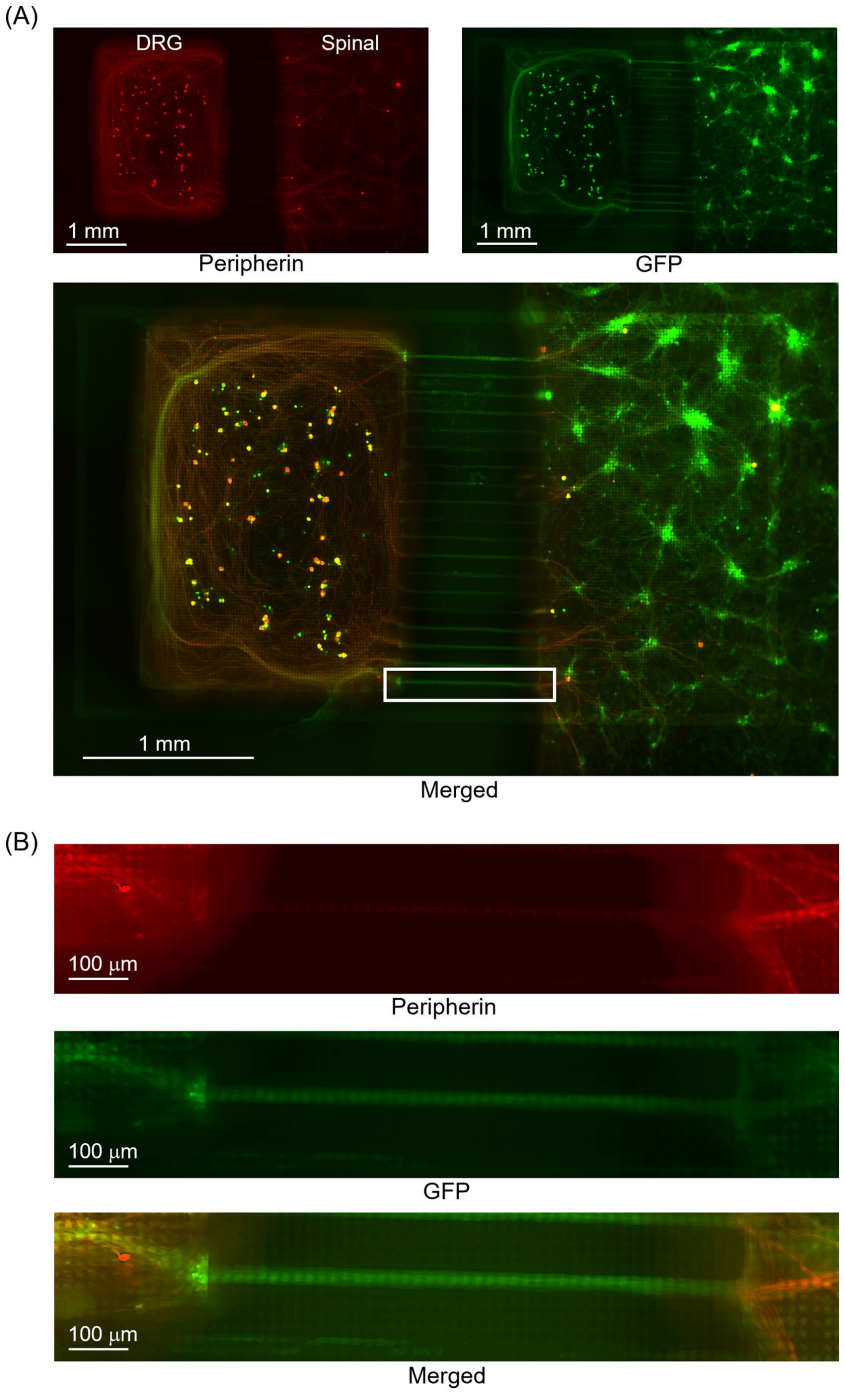
Immunofluorescence staining of co-cultured DRG and spinal neurons on an HD-MEA. **(A)** Overall view of the sample. **(B)** An enlarged view of the white-framed area in **(A)**. Peripherin+ neurons are depicted in red and NeuN+ neurons in green. Axons of Peripherin+ neurons that passed through the microtunnel extended across the culture area of spinal neurons. The presence of Peripherin+ and GFP + neurons indicated the possibility of selecting neurons for optical stimulation from the GFP fluorescence images. Green fluorescence protein, GFP.

Figure 4 presents the results of immunofluorescence staining of co-cultured DRG and spinal neuron samples on an HD-MEA. Figure 4B presents an enlarged view of the white-framed area in Figure 4A. While red indicates Peripherin+ areas, green indicates GFP + areas. GFP + areas show that the axons extended through the microtunnel, and Peripherin+ areas show that they covered the culture area of spinal neurons. This result confirms that DRG neurons successfully connected to spinal neurons located beyond the microtunnel exit. The presence of Peripherin+ and GFP + neurons indicated that it was feasible to select neurons for optical stimulation from the GFP fluorescence images. In addition, the results of immunofluorescence staining of NeuN in samples co-cultured on HD-MEA indicated the presence of 238 ± 34 neuronal nuclei per sample on the electrode unit.

### 3.2 Enhancement of spinal neuron activity via spinal transmission from DRG neurons

To assess the impact of DRG neuronal activity on spinal neuronal activity, maintaining minimal fluctuations in spinal neuronal activity during the experiment is essential. Furthermore, the input from the DRG neurons should induce a distinct increase in spinal neuronal activity compared to the state in which spinal neurons are directly stimulated to enhance their firing rate. Thus, we analyzed recorded signals to evaluate the effect of optical stimulation of DRG or spinal neurons on the firing rate of spinal neurons. First, we compared the results across the three experimental conditions to identify differences in the effect of activity transmission from the stimulation target on the increased activity of spinal neurons. Next, we assessed the cellular and network-level effects of activity transmitted from DRG neurons on the increased activity of spinal neurons.

For the two samples recorded in “DRG stim,” “SC stim,” and “control,” the number of waveform clusters increased by 2.1 and 3.2 folds for “DRG stim” and by 1.2 and 1.9 folds for “SC stim” compared to the “control,” respectively. Burst frequency increased both during and after stimulation under the “DRG stim” condition (in-stim, by 3.0 and 1.1folds; post-stim, by 1.1 and 1.1 folds) compared to the increase in burst frequency in the “control” (in-stim, by 0.7 and 0.7 folds; post-stim, by 0.7 and 0.5 folds). In the “SC stim,” burst frequency increased during stimulation, whereas it was similar to that in the “control” after stimulation (in-stim, 1.2- and 6.5-fold increases; post-stim, 0.5- and 0.8-fold increases). The results indicate that optical stimulation of DRG and spinal neurons increased the activity of spinal neurons. Moreover, stimulation of DRG neurons increased the post-stim burst frequency more than the stimulation of spinal neurons.

**Figure 5:**
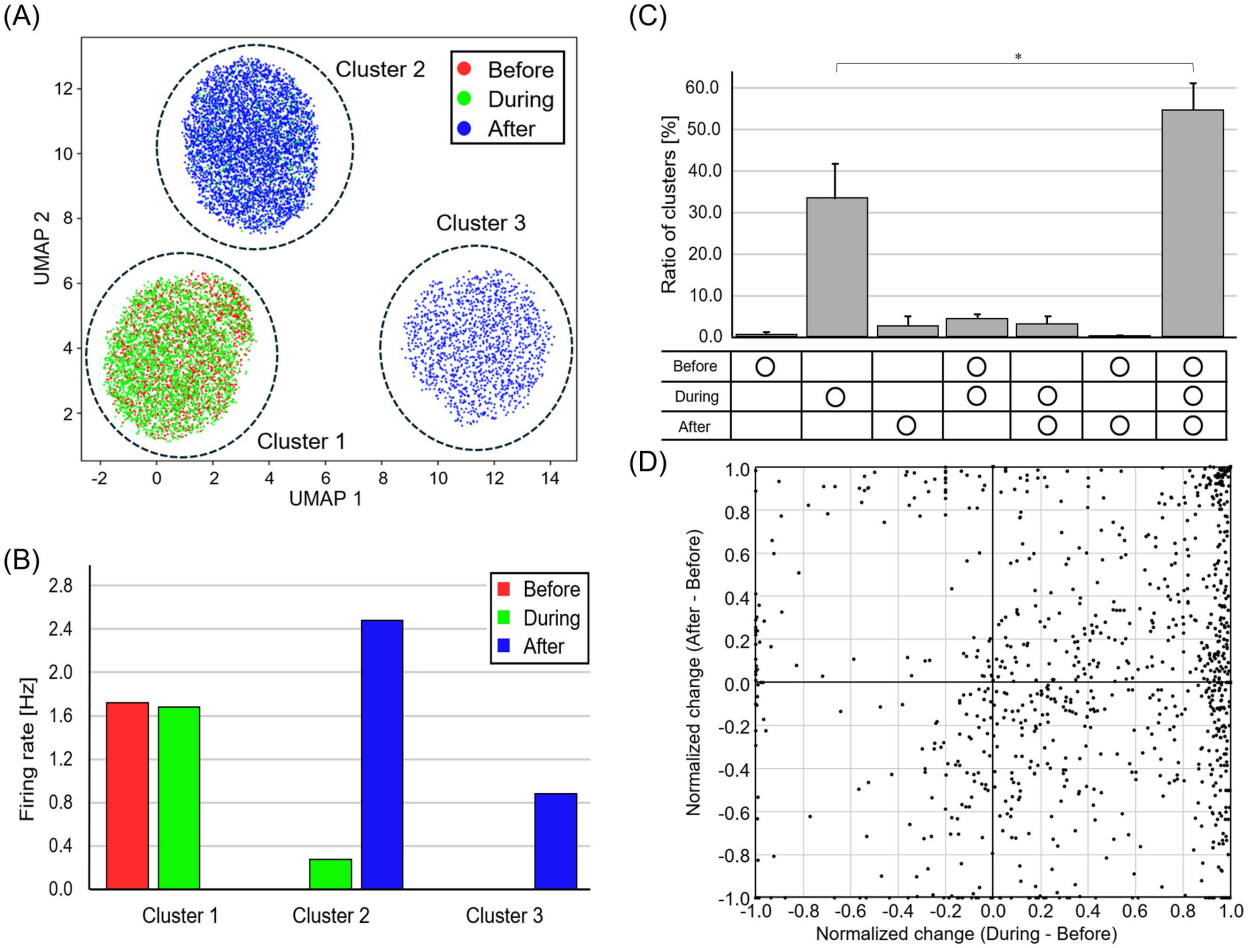
Change in firing rate for each waveform cluster. **(A)** Spike waveforms after dimensionality reduction and clustering. Red, green, and blue dots represent before, during, and after stimulation, respectively. **(B)** The temporal distribution of spikes contained in each waveform cluster. The unchanged waveforms of clusters 1 and 2 indicate consistent neuronal activity. **(C)** The temporal distribution of the ratio of the number of waveform clusters. Each bar represents the mean value, and the error bars indicate one standard deviation above the mean (*p < 0.05). Spinal neurons, which are normally inactive, become active upon receiving activity from DRG neurons. **(D)** Normalized change in firing rate during and after stimulation compared to before stimulation. Optical stimulation of DRG neurons enhanced the activity of spinal neurons.

We also evaluated the effect of optical stimulation of DRG neurons on the spontaneous activity of spinal neurons. Figure 5 presents the results of spike sorting from the signal recorded with a single electrode unit. In Figure 5A, each dot represents each spike, and the color represents the recording period (red, before stimulation; green, during stimulation; blue, after stimulation). Cluster 1 was recorded before and during the stimulation, cluster 2 during and after the stimulation, and cluster 3 after the stimulation (Figure 5B). The waveforms of clusters 1 and 2 did not change regardless of the presence or absence of stimuli, suggesting that the same neuron activity was recorded. The number of waveform clusters for the 5 samples used for both “DRG stim” and immunofluorescence staining was 217 ± 30, obtained by analyzing the signals for the entire recording period. Figure 5C presents the temporal distribution of the ratio of the number of waveform clusters. The ratio of clusters for waveform clusters consistently present across all recording times was significantly greater compared to those existing only during stimulation (*p* < 0.05, Wilcoxon signed-rank test). Because 38.3 ± 11.3% of the waveform clusters were not present before stimulation, it can be inferred that spinal neurons, which are normally inactive, become active upon receiving activity from DRG neurons. Spikes from 55.1 ± 9.7% of the waveform clusters were recorded throughout all recording times. For each waveform cluster, the plot in Figure 5D shows the normalized change in firing rate during and after stimulation compared to before stimulation. During stimulation, a substantial number of neurons exhibited elevated firing rate. In contrast, following stimulation, most neurons were unchanged in firing rate compared to the before stimulation period, although a subset exhibited either an increase or a decrease in the activity. The fact that many points are plotted in the region where both the vertical and horizontal axis values are greater than 0.0 indicates that optical stimulation enhanced the activity of spinal neurons.

Next, we evaluated the effect of optical stimulation of DRG neurons on the burst of spinal neurons. Burst frequency increased by 11.8 ± 17.8-fold during stimulation and by 7.9 ± 11.8-fold after stimulation. Optogenetic stimulation to DRG neurons led to more localized bursting activity in spinal neurons (Supplementary Figure 5). The average collective activity strengths were 41.3 ± 13.4% before stimulation, 30.9 ± 7.3% during stimulation, and 35.9 ± 8.7% after stimulation. While the Wilcoxon signed-rank test showed no significant differences, we observed a consistent trend: the average collective activity strength decreased in 7 out of 8 samples during stimulation and in 6 out of 8 samples after stimulation, compared to that before stimulation. This suggests that bursting became more localized during and after stimulation.

Analysis of the Pearson correlation coefficients between neuronal activities revealed that during and after stimulation, network synchronicity decreased, and localized bursts involving only a small number of neurons emerged. Compared to the correlation coefficients of before stimulation, those of during and after stimulation were 39.0 ± 13.8% and 80.6 ± 17.3%, respectively, indicating a decrease in overall network synchronicity during both periods.

**Figure 6:**
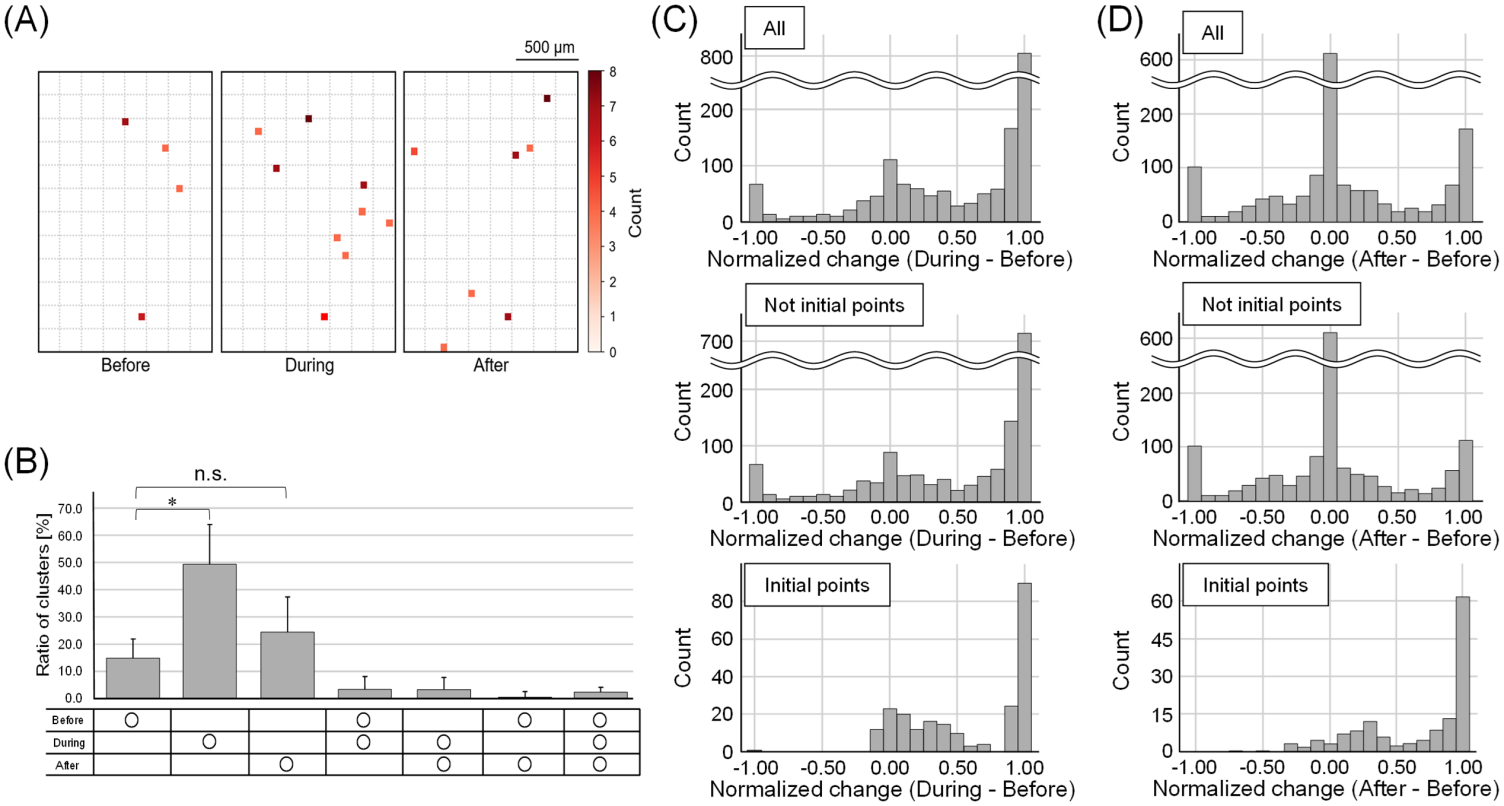
Change in the number of burst initiation points and their firing rate. **(A)** The electrode units that were the initiation points of bursts before, during, and after stimulation. The dotted squares indicate intervals of 10 electrodes. The darker the red color, the more times the burst started from the units. The number of burst initiation points increased during and after stimulation. **(B)** The temporal distribution of the ratio of the number of waveform clusters that were burst initiation points. Each bar represents the mean value, and the error bars indicate one standard deviation above the mean (*p < 0.05; n.s., not significant). **(C,D)** Histograms showing normalized changes in firing rates of waveform clusters that were burst initiation points **(C)** during and **(D)** after stimulation, relative to the before stimulation period. The peak near 1 represents spinal neurons that were inactive before stimulation but became active in response to DRG stimulation. The peak near 0 corresponds to neurons whose firing rates remained constant regardless of stimulation. During stimulation, firing rates increased in both non-burst-initiating and burst-initiating clusters. After stimulation, most non-burst-initiating clusters showed little to no change in firing rate, whereas burst-initiating clusters exhibited elevated firing rates.

We also observed changes in burst initiation points. Figure 6A shows the electrode units that were the initiation points of bursts before, during, and after stimulation. The number of burst initiation points increased by 5 ± 2 during stimulation and 2 ± 2 after stimulation compared to before stimulation. Bursts originating from distinct initiation points exhibited different propagation patterns, whereas bursts originating from the same initiation point tended to show similar propagation patterns before and after stimulation (Supplementary Figure 6).

We then identified the waveform clusters initiating the bursts. The proportion of burst-initiating waveform clusters was significantly greater during stimulation compared to that in the period before stimulation (*p* < 0.05, Bonferroni-corrected Wilcoxon signed-rank test; Figure 6B). Although the proportion after stimulation was not significantly different from that before stimulation, it tended to be greater (*p* > 0.05, Bonferroni-corrected Wilcoxon signed-rank test). Additionally, 2.7 ± 2.6% of the waveform clusters were the common burst initiation points throughout all recording times. Figure 6C presents histograms of normalized firing rate changes for each waveform cluster during stimulation, whereas Figure 6D illustrates the corresponding changes after stimulation. The peak near 1 represents spinal neurons that were inactive before stimulation but became active in response to DRG stimulation. The peak near 0 corresponds to spinal neurons that maintained a consistent level of activity regardless of stimulation. During stimulation, firing rates increased in both non-burst-initiating and burst-initiating clusters. After stimulation, most non-burst-initiating clusters showed little to no change in firing rate, whereas burst-initiating clusters exhibited elevated firing rates. Notably, not only did previously inactive neurons become active in response to stimulation, but previously active neurons also increased their firing rates and began to lead synchronized bursts.

Finally, to investigate the role of inhibitory neurons, we optically stimulated DRG neurons in a neural network where input from inhibitory spinal neurons was blocked. In this condition, all burst initiation points were preserved before and after stimulation (Supplementary Figure 7), suggesting a role for inhibitory neurons in modulating burst initiation point dynamics under normal conditions.

## 4 Discussion

In the present study, we developed a co-culture system using DRG and spinal neurons. The axons of DRG neurons passed through the microtunnels connecting the separate culture areas, forming functional connections with spinal neurons. Immunofluorescence staining confirmed the successful separation of DRG and spinal neurons with high accuracy. The observed sustained increase in burst frequency following DRG neuron stimulation suggests DRG-induced plasticity within the spinal neuronal network. By analyzing protein expressions in monocultured and co-cultured samples, we showed that the proposed co-culture method can spatially separate DRG neurons from spinal neurons. Notably, the low cross-contamination rate between DRG and spinal neurons achieved in this study demonstrates the validity and reliability of our co-culture approach for investigating neuron-specific interactions in sensory pathways.

We showed that the frequency of spinal neuron activity can be increased through activity transmission induced by optical stimulation. Individual waveform clusters represent the activity of individual spinal neurons. The number of neuron nuclei on electrode units is approximately equal to the number of waveform clusters, suggesting that activity can be recorded from almost all neurons. In other words, the proposed method enables the recording of activity from individual neurons across the entire network, which is not feasible with the existing methods. The number of waveform clusters with enhanced activity and burst frequency increased during stimulation. These results are consistent with existing findings of activity transmission from DRG neurons or spinal neurons via the excitatory neurotransmitter glutamate (Dubin and Patapoutian, 2010), as well as studies indicating that increased activity of neurons facilitates spatiotemporal synchronization of activity (Contreras-Hernández et al., 2015). In the present study, the number of waveform clusters with an increased firing rate after stimulation was greater in the “DRG stim” than before stimulation under the “SC stim.” Moreover, burst frequency under the “SC stim” was similar to that of the “control,” whereas it increased under the “DRG stim.” These results suggest that activity transmission from DRG neurons to spinal neurons is more effective in increasing the frequency of spinal neuronal activity than activity transmission between spinal neurons. As spinal neurons contain both inhibitory and excitatory neurons (Sullivan and Sdrulla, 2022), the firing rate of the coupled neurons does not increase if the optical stimulation is delivered to spinal inhibitory neurons. The differing trend in burst initiation point changes, depending on the presence or absence of inhibitory input, suggests that inhibitory neurons regulate the synchronization of the neuronal network. In addition, neuropeptides, such as CGRP, released by DRG neurons at the synapse depolarize the membrane potential of spinal neurons (Jang and Garraway, 2024), facilitating the induction of neuronal activity. Therefore, the stimulation of DRG neurons using the proposed method is useful for reproducing the phenomenon of activity transmission from DRG neurons to increase the frequency of spinal neuronal activity in the culture system.

This study identified key changes in the activity patterns of burst-leading spinal neurons induced by activity propagation from DRG neurons. Specifically, we found that DRG-derived activity causes diverse alterations in the firing properties of burst-leading spinal neurons and leads to sustained modifications in network burst dynamics. The following sections describe these two major findings in detail: the diversity in activity changes among burst-leading spinal neurons, and the persistence of the altered network activity.

First, burst-leading spinal neurons exhibited diverse changes in activity in response to DRG stimulation. Among all burst-leading neurons, 74.5% had been inactive prior to stimulation but became active. Additionally, 7.3% of burst-leading neurons were already active before stimulation and showed increased firing rates. These results suggest that activity transmitted from DRG neurons induces both the recruitment of previously inactive neurons and the enhancement of activity in spontaneously active neurons to lead network bursts. During stimulation, burst frequency increased mainly due to the recruitment of previously inactive neurons, whereas after stimulation, this increase was induced by both newly activated neurons and those with heightened pre-existing activity. Previous observations indicated that DRG neurons connect to specific spinal neurons (Wu et al., 2021) and that burst-leading neurons exist in the neuronal network of the central nervous system (Kobayashi et al., 2024). Based on these findings, an increase in the burst initiation points following DRG stimulation suggests that DRG neurons connect to specific spinal neurons capable of initiating bursts, and activity transmission from DRG neurons induced activity in these neurons, which are inactive or active spontaneously. This emphasizes the cell-specific activity transmission capacity of DRG neurons. Based on the results of the temporal correlation between neuronal activities, the inability to maintain network synchronicity can likely be attributed to widespread activity inhibition caused by the activation of inhibitory spinal neurons. Considering that bursts can alter the excitation-inhibition balance (Suresh et al., 2016), the observed changes in burst size are likely attributable to the activation of inhibitory neurons.

Second, the increased burst frequency in the spinal neuronal network persisted for at least 20 min after DRG stimulation. The previous study (Russo and Hay, 2023) showed that the CGRP released from DRG neurons can diffuse beyond synapses between DRG and spinal neurons (volume transmission), and induce excitation of surrounding spinal neurons. It has also been reported that CGRP-induced neuronal hyperexcitability can persist for several hours (Li et al., 2019). Therefore, it is suggested that neuropeptide release from DRG neurons facilitates sustained depolarization of both burst-leading and neighboring spinal neurons, thereby promoting burst generation. In addition to volume transmission of neuropeptides, it is also possible that short-term and long-term potentiation at synapses between spinal neurons contributed to the enhanced activity of spinal neurons. Our experimental results demonstrate that activity transmission from DRG neurons effectively increases the firing rate of spinal neurons, affecting the activity properties of the spinal neuron network. This increased activity transmission from spinal neurons to the brain is likely to alter neuronal processing in the thalamus and cortex, potentially contributing to the development of chronic pain.

A limitation of the current method is the inability to identify individual spinal neurons directly connected to specific DRG neurons. Consequently, assessing how particular DRG neuron subtypes differentially influence specific spinal neuron populations remains challenging. Integrating cell-type identification methods—such as spatial transcriptomics or functional connectivity analyses based on neuronal activity correlations (Gyllborg et al., 2020; Kobayashi et al., 2019)—into future iterations of our co-culture platform could overcome this limitation. Such improvements would greatly enhance our understanding of the precise neuronal circuits involved in nociceptive signal transmission. Future studies could integrate fluorescent labeling and functional connectivity analyses to clarify specific DRG-spinal and spinal-spinal neuronal interactions. Combining these approaches with spatial transcriptomics could significantly advance our understanding of sensory processing circuits.

Applying our proposed method can further elucidate *in vivo* phenomena. In the living body, chemical substances like capsaicin bind to receptors expressed on DRG neurons, thereby inducing their activity. Local pharmacological stimulation of DRG neurons would allow us to replicate stimulus-specific activity transmission. While the current device features a small culture chamber for DRG neurons, smaller in size than the 1.6 mm tip of a commonly used 200 μl pipette (Supplementary Figure 8), modifying the shape of the PDMS microstructures would enable the construction of an experimental system capable of repeated, localized chemical stimuli. Furthermore, in the living body, DRG neuronal input is processed in the spinal cord, and spinal neurons transmit activity to higher central nervous systems like the thalamus. By increasing the number of culture chambers and co-culturing neurons from higher central nervous systems, we could uncover the mechanisms of sensory information processing in the brain.

In conclusion, we constructed an experimental system in which DRG and spinal neurons were co-cultured on HD-MEAs. The activity of individual DRG neurons was controlled through optical stimulation, and increased activity transmission from DRG neurons to spinal neurons elevated the frequency of spinal neuronal activity. The proposed co-culture system offers a robust platform to explore the cellular mechanisms underlying the sensory neuron-driven modulation of spinal neuronal networks, with broad implications for understanding pathological conditions such as chronic pain.

## Data Availability

The raw data supporting the conclusions of this article will be made available by the authors, without undue reservation.
