# Supplementary material for: Sensory-spinal neuron co-culture platform enables analysis of sensory-driven spinal activation
Source: Front Neurosci. 2025 Jul 7;19:1619340. doi: 10.3389/fnins.2025.1619340 (PMC12277374; doi:10.3389/fnins.2025.1619340)
Supplement: Supplementary file 1 [file Table_1.DOCX]

Supplementary Material

# Supplementary Figures and Tables

## Supplementary Figures


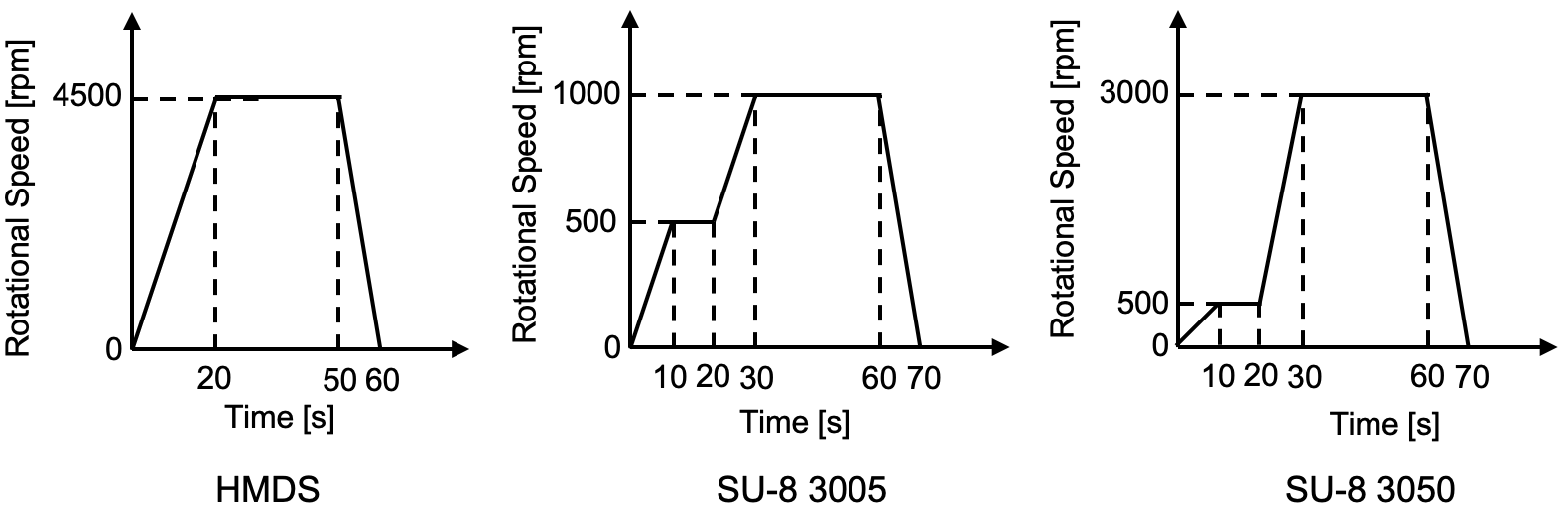


**Supplementary Figure 1.** Parameters of spin-coating.


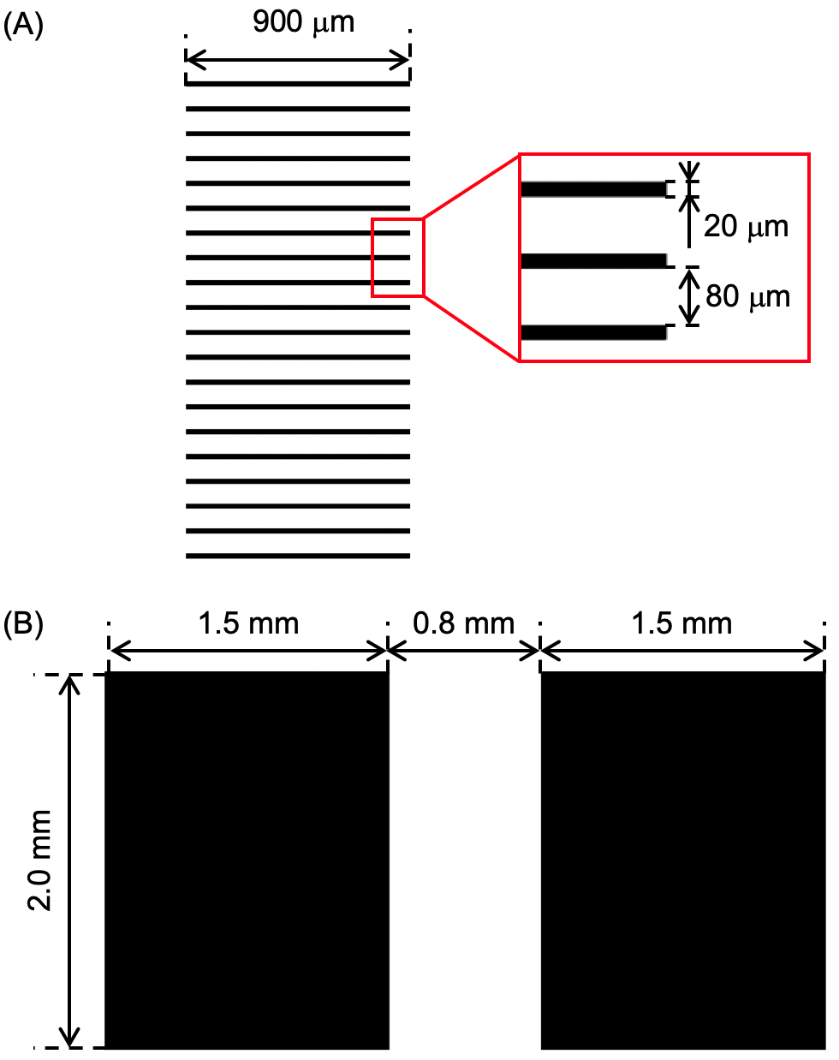


**Supplementary Figure 2.** Exposure patterns of the SU-8 molds. (A) Shape of the microtunnel mold; 20 microtunnels were made in rectangular patterns. (B) Shape of the culture chamber mold; 2 chambers were made in rectangular patterns.


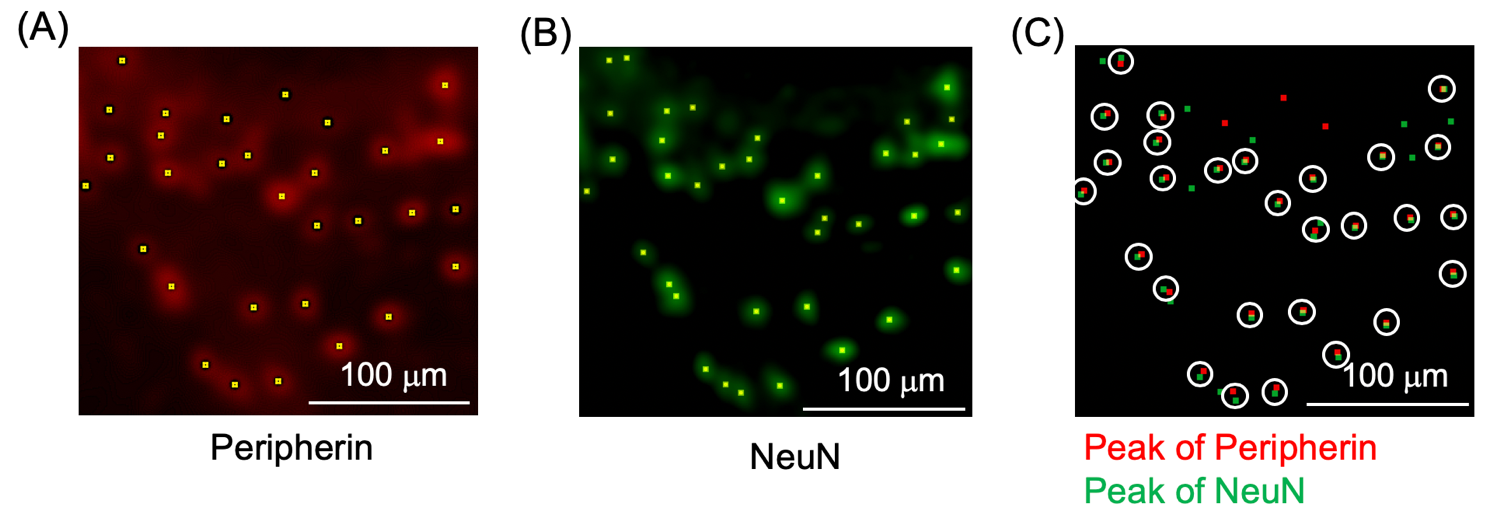


**Supplementary Figure 3.** Peak Detection in Immunofluorescence Staining of DRG Neurons. The Peripherin+ areas are shown in red (A), while the NeuN+ areas are shown in green (B). Brightness peaks are depicted in yellow in both (A) and (B). (C) Identification of Peripherin+/NeuN+ neurons. Peaks in the Peripherin+ regions are indicated in red, and those in the NeuN+ regions are indicated in green. White-circled areas containing both red and green peak points represent Peripherin+/NeuN+ neurons. Peripherin+/NeuN+ neurons were identified based on the distance between the peaks.


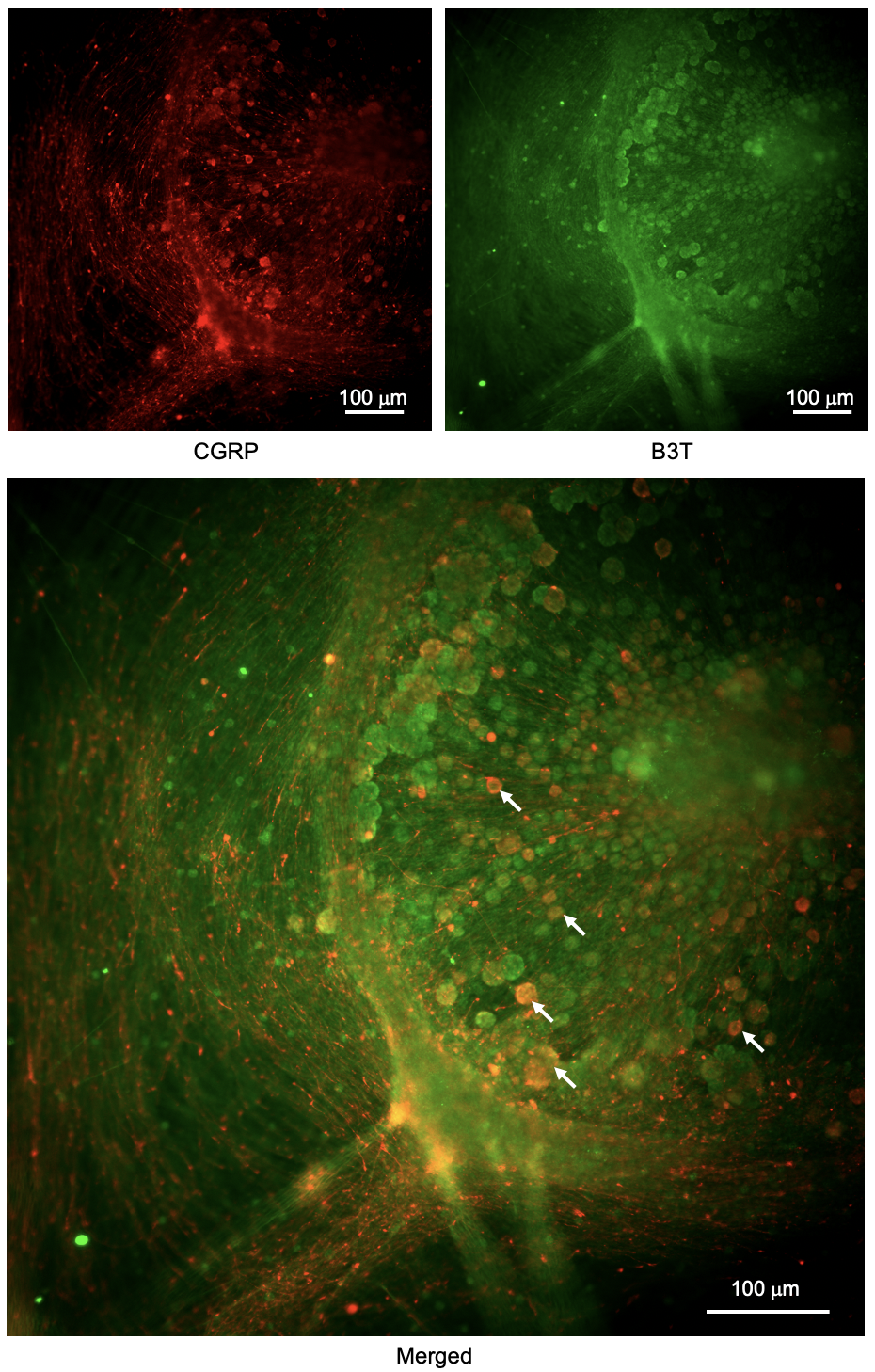


**Supplementary Figure 4.** Immunofluorescence staining of DRG neurons. CGRP+ neurons are indicated in red, and B3T+ neurons are depicted in green. A total of 10.8% of DRG neurons were positive for CGRP.
